# Antipsychotic Prescribing in VA-Contracted Community Nursing Homes and Incident Use Among Veterans

**DOI:** 10.1093/geroni/igab046.1286

**Published:** 2021-12-17

**Authors:** Patience Moyo, Emily Corneau, Portia Cornell, Amy Mochel, Kate Magid, Cari Levy, Vincent Mor

**Affiliations:** 1 Brown University School of Public Health, Providence, Rhode Island, United States; 2 Providence VA Medical Center, Providence, Rhode Island, United States; 3 Rocky Mountain VA Medical Center, Aurora, Colorado, United States; 4 Brown University, Providence, Rhode Island, United States

## Abstract

The Veterans Health Administration (VA) is increasingly purchasing long-term care for eligible Veterans from non-VA, community nursing homes (CNHs). Antipsychotics present safety risks for older adults, but it is unknown how the prevalent use of antipsychotics at CNHs influences whether newly admitted Veterans will initiate antipsychotic therapy. This study used 2013-2016 VA data, Medicare claims, Nursing Home Compare, and Minimum Data Set (MDS) assessments. We identified 10,531 long-stay CNH episodes for Veterans not prescribed antipsychotics 6 months before CNH admission. We categorized Veterans by whether, 12 months before admission, they were diagnosed with FDA-approved indications (including schizophrenia, Tourette’s syndrome, Huntington’s disease) for antipsychotic use. The exposure was the proportion of all CNH residents prescribed antipsychotics in the quarter preceding a Veteran’s admission. Using adjusted logistic regression, we analyzed two outcomes measured using MDS assessments collected ~100 days after CNH admission: 1) new antipsychotic use, and 2) new diagnosis for an FDA-approved indication among Veterans without these conditions at admission. Among antipsychotic-naïve Veterans admitted to CNHs, 7,924 (75.2%) lacked an antipsychotic indication. Prevalent antipsychotic use in CNHs ranged 0%-10.9% (quintile 1) and 25.7%-91.4% (quintile 5). The odds of initiating antipsychotic use increased with higher CNH antipsychotic use rates (OR=2.52, 95% CI:2.05-3.10, quintile 5 vs. 1), as did the odds of acquiring a new indication (OR=2.08, 95% CI:1.27-3.40, quintile 5 vs. 1). Provider practices may be influencing new diagnoses indicating antipsychotic use at CNHs with high antipsychotic use. It may be important for VA to consider antipsychotic use when contracting with CNHs.

